# PotatoLeafNet: two-stage convolutional neural networks for effective Potato Leaf disease identification and classification

**DOI:** 10.3389/frai.2025.1668839

**Published:** 2026-01-12

**Authors:** Girigula Durga Bhavani, Mukkoti Maruthi Venkata Chalapathi

**Affiliations:** School of Computer Science and Engineering (SCOPE), VIT-AP University, Amaravati, Andhra Pradesh, India

**Keywords:** potato leaf diseases, convolutional neural networks, dual CNN, sequential image augmentation, early blight

## Abstract

**Introduction:**

Potato foliar diseases, particularly early and late blight, pose a serious threat to yield and food security, yet reliable visual recognition remains challenging due to cultivar heterogeneity, variable symptom expression, and acquisition noise in field-like imagery. To address these issues, we propose PotatoLeafNet, a two-stage deep learning framework that combines a fixed-sequence image-augmentation pipeline with a compact, task-optimized 11-layer convolutional neural network (CNN) using 3 × 3 kernels for robust, data-efficient classification of potato leaf conditions (Healthy, Early Blight, Late Blight).

**Methods:**

We construct a dataset of 4,072 labeled potato leaf images from the PlantVillage-Potato subset and standardize all inputs to 224 × 224 RGB tensors with pixel intensities normalized to [0,1]. A balanced, fixed-order augmentation policy—comprising rotation, translation, shear, zoom, horizontal flipping, brightness adjustment, and channel jitter—is applied exclusively to the training split, increasing it to 6,000 images (2,000 per class) while keeping the validation and test sets free of synthetic samples. The second stage consists of an 11-layer CNN implemented in TensorFlow/Keras and trained with categorical cross-entropy loss and the Adam optimizer under a unified training and evaluation protocol. Performance is benchmarked against strong CNN and hybrid baselines, including ResNet-50 + VGG-16, VGG-16 + MobileNetV2, MobileNetV2, and Inception-V3.

**Results:**

On the PlantVillage-Potato test set, PotatoLeafNet achieves 98.52% accuracy, 98.67% macro-precision, 99.67% macro-recall, 99.16% macro-F1, and 1.00 macro-AUC, outperforming all baseline models under identical preprocessing and training conditions. In particular, PotatoLeafNet surpasses ResNet-50 + VGG-16 (97.10% accuracy, AUC 0.98), VGG-16 + MobileNetV2 (94.80% accuracy, AUC 0.93), MobileNetV2 (93.20% accuracy, AUC 0.92), and Inception-V3 (92.50% accuracy, AUC 0.91). Short 10-epoch runs yield stable convergence (training accuracy 88.22%, validation accuracy 86.91%, test accuracy 88.15%), indicating efficient learning from the augmented distribution.

**Discussion:**

The results demonstrate that explicitly coupling a fixed sequential augmentation stage with a lightweight 3×3-kernel CNN enables high tri-class accuracy, strong recall for disease classes, and improved generalization relative to deeper or fused architectures, without incurring substantial computational cost. By emphasizing disease-relevant structure while limiting overfitting, PotatoLeafNet provides a practical and resource-efficient solution for automated screening of potato leaf health in real-world agronomic settings, supporting timely and data-driven disease management.

## 1 Introduction

The potato (*Solanum tuberosum*) is a staple crop and a vital source of calories and micronutrients for millions of people worldwide (Sunjoyo and Nugroho, 2022). Yields, however, are highly vulnerable to foliar pathologies most notably early blight and late blight which inflict substantial economic losses and threaten local food security when outbreaks go undetected or unmanaged (Hou et al., 2021). Early, reliable diagnosis is therefore essential for timely intervention, yet traditional field scouting by experts is labor-intensive, subjective, and difficult to scale across heterogeneous environments and planting cycles. These practical constraints motivate automated systems that deliver accurate, consistent, and rapid decisions directly from visual evidence. Convolutional neural networks (CNNs) have reshaped image understanding by learning hierarchical representations from data and have already shown strong performance in plant disease recognition tasks (Tugrul et al., 2022). Despite this promise, potato leaf disease classification remains challenging in real deployments. Symptoms vary with cultivar, phenological stage, and stress conditions; image capture occurs under fluctuating illumination, background clutter, motion blur, and sensor noise; and publicly available datasets are often limited in size and balance across classes. Moreover, many prior approaches emphasize single-disease detection rather than precise multi-class discrimination among healthy leaves and the major disease categories (early blight and late blight) required for agronomic decision-making (Alhammad et al., 2025). These factors collectively degrade generalization and complicate robust deployment on resource-constrained devices.

To address these limitations, we propose PotatoLeafNet, a two-stage convolutional framework for potato leaf disease detection and classification that explicitly couples data diversification with a compact, task-optimized classifier. Stage 1 performs sequential image augmentation including rotations, scalings, flips, and related geometric and photometric transforms to expand the training distribution and encode invariances that mirror field variability (Potato Leaf Disease Dataset, 2025). By structuring augmentation as a dedicated stage, the pipeline intentionally exposes the learner to controlled perturbations that emulate acquisition noise and viewpoint change, thereby improving robustness without inflating model capacity. Stage 2 is a lightweight CNN tailored for potato leaves: convolutional blocks with ReLU activations and 3 × 3 kernels extract localized texture and lesion-edge cues; max-pooling progressively reduces spatial resolution while preserving salient patterns; global average pooling compacts feature maps to mitigate overfitting; and a fully connected head (a 128-unit ReLU layer followed by a softmax) produces calibrated class probabilities for healthy, early blight, and late blight. This design emphasizes parameter efficiency and computational tractability while retaining discriminative power under real-world noise. Our evaluation plan reflects these deployment goals. We train and test PotatoLeafNet on a diversified collection of potato leaf images spanning healthy, early blight, and late blight categories (Sangar and Rajasekar, 2025). Performance is assessed using standard metrics accuracy, precision, recall, and F1-score to quantify both overall correctness and class-wise reliability. We further benchmark against contemporary CNN-based methods to examine accuracy efficiency trade-offs and to determine whether an explicit augmentation stage coupled with a compact classifier offers practical advantages over monolithic architectures. In addition, we analyze error modes to illuminate failure cases (e.g., tiny lesions with blurred boundaries, confounding background textures), informing future improvements to both model and data regimen. The significant contributions to the research are:

- Introduced a novel two-stage convolutional neural network architecture, PotatoLeafNet, specifically optimized for high accuracy in detecting and classifying potato leaf diseases, addressing limitations in existing models
- Implemented advanced sequential image augmentation techniques within a CNN framework to significantly enhance the model’s ability to generalize across diverse and unseen environmental conditions, a step beyond traditional augmentation practices.
- Conducted a rigorous evaluation of the PotatoLeafNet model using an extensive dataset that includes a balanced representation of Healthy, Early Blight, and Late Blight potato leaf images, ensuring robust testing against varied disease manifestations.
- Demonstrated superior performance of the PotatoLeafNet model through a comparative analysis with existing state-of-the-art models, highlighting advancements in accuracy and computational efficiency.

The study introduces a two-stage CNN-based potato leaf disease detection and classification method. Deep learning and image augmentation increase illness detection using this method.

## 2 Basic preliminaries and related research work

Leaf diseases are a common problem in plants and crops, and they can cause significant damage to both the yield and quality of the harvest various factors cause leaf maladies including fungi, bacteria, viruses, and environmental stressors. Depending on the specific disease and the plant species affected, the symptoms of leaf diseases can vary greatly. Some common symptoms of leaf diseases include discoloration, spotting, wilting, deformity, and defoliation (Afzaal et al., 2021). The entire plant may sometimes be involved, leading to stunted growth, reduced yield, or even death. Detecting and classifying diseases is significant for the control of the conditions. It can be done using various techniques such as visual inspection, laboratory analysis, and remote sensing. Recently, methods for automatically identifying and classifying leaf diseases using images of plant leaves have also been developed utilizing machine learning and computer vision techniques.

### 2.1 Types of potato leaf diseases

**Figure 1:**
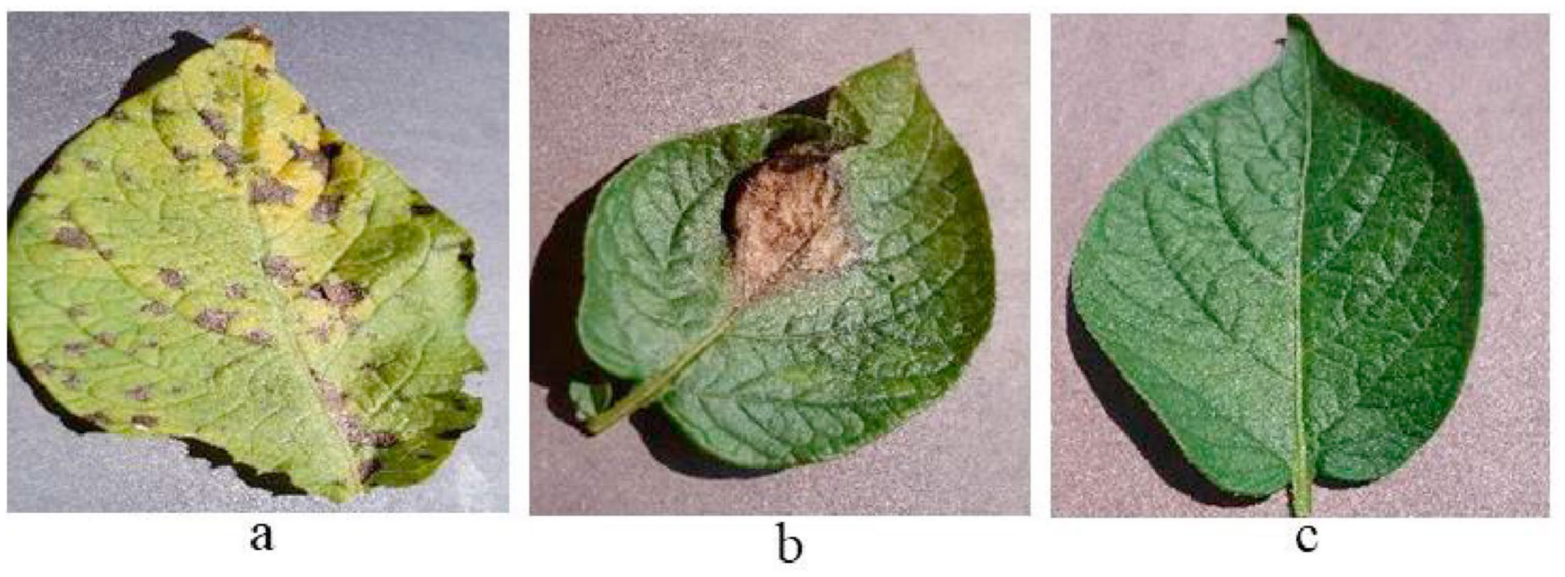
Sample images **(a)** potato early blight, **(b)** potato late blight, **(c)** potato healthy leaf.

Potato foliage is vulnerable to a spectrum of pathogens with markedly different epidemiologies and symptomatology. Late blight (*Phytophthora infestans*), a fast-spreading oomycete disease, remains the most devastating, initiating water-soaked lesions that rapidly coalesce into necrotic brown areas with a characteristic grayish, downy sporulation under humid conditions, and frequently extending to tubers (Jafar et al., 2024). Early blight (Alternaria solani) typically emerges on older leaves as discrete dark lesions that enlarge with concentric “target-spot” rings, progressing to chlorosis and premature defoliation in warm, humid environments (Potato Disease Types, 2025). Viral diseases such as Potato virus Y (PVY) and Potato leafroll virus (PLRV), both primarily aphid-borne, induce mosaic mottling, leaf curling, and canopy yellowing (PVY), or the diagnostic upward rolling and brittle texture of leaves (PLRV), with attendant losses in yield and tuber quality that depend on cultivar and viral strain (Liu and Wang, 2021). Soil and vascular-invading fungi drive wilt syndromes early dying dominated by Verticillium dahliae and late dying associated with Fusarium spp. leading to progressive wilting, chlorosis, and necrosis that culminate in significant productivity declines. Bacterial threats such as bacterial ring rot cause leaf yellowing, wilting, and vascular browning with corky ring formation, and can persist in soils and on equipment, complicating eradication efforts. While multiple leaf diseases impair crop performance, early and late blight are generally the most consequential for field management decisions; representative phenotypes for Early blight, Late blight, and Healthy leaves are shown in Figure 1.

### 2.2 Literature review on potato leaf disease detection and classification

This potato leaf disease detection and categorization literature review summarizes current methodologies. Radwan et al. (2025) developed a weather-driven pipeline for early and late blight using K-means, PCA, copula analysis and multiple classifiers, with binary Greylag Goose Optimization for feature selection. On a 4,000-record meteorological dataset, the best MLP with selected features reached 98.3% accuracy. This tabular risk-forecasting setup complements image-based screening. Chen and Liu (2025) introduced CBSNet with Channel Reconstruction Multi-Scale Convolution and Spatial Triple Attention, plus a Bat–Lion training strategy for robustness. On a self-built potato leaf image set, CBSNet achieved 92.04% accuracy and 91.58% precision, extracting tiny lesions and blurred edges effectively. Dey et al. (2025) proposed a lightweight CNN tailored for real-time classification, reducing depth and parameters to 204,227 while preserving accuracy on high-resolution potato leaf images. The model attained 98.6% test accuracy and class-wise precision of 0.99 (early blight), 0.98 (late blight), 1.00 (healthy), outperforming VGG16, AlexNet, and ResNet50. Sinamenye et al. (2025) fused EfficientNetV2-B3 with a Vision Transformer to couple local convolutional features with global context. Trained on the Potato Leaf Disease Dataset reflecting field variability, the hybrid reached 85.06% accuracy, improving prior results by 11.43 points. Salihu et al. (2025) built a CNN trained with Adam, using scaling, augmentation, and normalization over a curated set of healthy, early blight, and late blight images. The model achieved 96.88% accuracy, with class metrics including precision 0.76, recall 0.93, F1 0.84 for healthy and near-perfect scores for blight classes. Ala’a (2025) extracted generalized Jones polynomial texture features and classified with SVM on Plant Village potato images. The GJP-SVM pipeline preprocessing, feature extraction, dimensionality reduction, classification reached 98.45% accuracy, showing strong performance from hand-crafted descriptors. Zhang et al. (2025) benchmarked VGG16, MobileNetV1, ResNet50, and ViT, then proposed VGG16S with global average pooling, CBAM attention, and Leaky ReLU to shrink parameters to 15 M. After response-surface hyperparameter tuning, VGG16S achieved 97.87% test accuracy and generalized well on public sets. Kaur et al. (2025) presented PotConvNet, a compact CNN trained on two potato image datasets with resizing, normalization, augmentation, and fixed splits. Reported accuracies were 99.76% (Dataset 1) and 97.78% (Dataset 2), validated by F1, precision, recall, Cohen’s kappa, and ROC AUC. Nur et al. (2025) optimized Inception V3 via transfer learning and targeted fine-tuning of terminal layers on a domain-specific potato leaf set. The approach yielded 97.78% accuracy with precision 98%, recall 98%, F1 98%, offering strong performance with practical efficiency. Shah et al. (2025) introduced PLDC-Net, using EfficientNet-B1 as a backbone, fine-tuned with dense layers and an SVM output head; data balancing and augmentation were emphasized. On an unseen test set, the model achieved 98.39% average accuracy, providing a reliable transfer-learning baseline for multi-disease identification. We diagnose and categorize potato plant diseases. Various studies on diagnosing and categorizing potato plant diseases may be found in the literature on potato leaf disease classification and detection (Fuentes et al., 2017). CNN and other deep learning approaches have shown promise for automating the detection and classification process, reducing the need for human expertise-several CNN architectures, transfer learning, feature extraction, and ensemble methods to improve accuracy and robustness (Geetharamani and Pandian, 2019). The study (Ahmed et al., 2025) suggested a deep CNN model to identify outstanding and ailing foliage across crops. They trained their model using the Plant Village dataset, which includes photos of diseased and healthy leaves and the backgrounds of 38 distinct crop kinds. However, they did not zero in on potato crop illnesses, and the data used to prepare the algorithm in the United States and Switzerland missed Pakistan-endemic infections on potato leaves.

Despite having little data, the scientists used deep learning, specifically CNNs, to identify potato illnesses (Lee et al., 2020). A CNN model was created (Awal et al., 2019) to distinguish between healthy potato leaves and those affected by early or late blight. They also used the regionally targeted Plant Village dataset in their research (Khalifa et al., 2021). In this research, we looked at how well deep learning methods and convolutional neural networks, in particular, might do at identifying diseases on potato leaves. The authors trained a CNN network using a collection of photos of diseased potato leaves. The success of the suggested method in illness detection demonstrates the promise of deep learning for this application area. According to Ghosal et al. (2019), the CNN model has the ability to differentiate between various plant classes. This study, Rathod et al. (2020) used deep learning to detect potato leaf blight early. The authors trained a CNN architecture to interpret potato leaf images. The model’s early blight detection highlights deep learning’s potential for potato leaf diseases. The authors examined deep learning and transfer learning for potato disease diagnosis (Liang et al., 2019). Using potato leaf images, the authors updated VGG16, a pre-trained CNN model. Pre-trained CNN models with transfer learning were useful in potato disease detection. A network for identifying and assessing plant diseases was demonstrated in Ferentinos (2018). To distinguish between healthy and diseased plants from photographs of their leaves (Rozaqi et al., 2020; Sanjeev et al., 2020) looked at many deep-learning architectures. These included AlexNet, Overfeat, AlexNetOWTBn, VGG, and GoogLeNet. The authors applied transfer learning to the PlantVillage dataset to identify local agricultural diseases. We developed a CNN model to detect potato plants with early, late, or robust blight. We trained the model using PlantVillage, disease data. FFNNs can distinguish between early, late, and healthy foliage (Barman et al., 2020). They trained and tested their system using PlantVillage. Using a self-built CNN (SBCNN) model, Tiwari et al. (2020) classified potato leaves as early, late, or healthy. The regional PlantVillage dataset improved their model’s accuracy. They did not utilize experimental data to validate their model. Gupta et al. (2019) extracted and classified features using KNN, SVM, a neural network, and a pre-trained VGG19 model using KNN, SVM, and a neural network. PlantVillage has trained the computer to identify early and late blight symptoms on potato foliage. Research demonstrates that CNNs and other forms of deep learning effectively identify and categorize diseases in potato leaves. To further improve the performance of deep learning models, even with minimal training data, practitioners have turned to methods including data augmentation, transfer learning, and fine-tuning pre-trained models. These findings show that deep learning may improve potato disease detection and classification, which is crucial for the crop’s long-term health.

### 2.3 Literature on potato leaf disease detection and classification using augmentation and deep learning models

The study, Bappi et al. (2025) provided a novel deep-learning algorithm for potato leaf tissue disease detection using augmentation approaches. Scaling, flipping, and rotating the training dataset enhanced the model’s accuracy. The research (Rahman et al., 2021) examined how different kinds of enhancement may affect deep Convolutional Neural Networks (CNNs) ability to spot illnesses in potato leaves. In this research (Plant Village Dataset, 2024), we applied deep learning models and data augmentation to improve our ability to identify diseases in potato leaves. The authors in the research work developed a deep learning-based method that uses data augmentation techniques to detect potato diseases. They used augmentation methods, including scaling, flipping, and rotating, to upsurge the size of the training dataset. In potato disease identification, training a CNN model on the expanded dataset resulted in high accuracy. Table 1 summarizes augmentation and deep learning studies on potato leaf disease detection and classification. These studies demonstrate the scope of current potato leaf disease identification and categorization efforts. While typical machine learning methods have shown promise, recent research has demonstrated that deep learning, particularly CNNs, may boost accuracy and automation. The proposed study on two-stage PotatoLeafNet CNN architectures will examine their ability to accurately identify and classify potato leaf diseases.

**Table 1:** Summary of the literature on potato leaf disease detection and classification using augmentation and deep learning models.

Reference no	Approach	Data augmentation methods	Deep learning model	Key findings
Khalifa et al. (2021)	Deep learning-based approach	Random cropping, flipping, and rotation	CNN	Achieved 94% accuracy, demonstrating robustness in varied conditions
Ghosal et al. (2019)	Deep learning with augmentation techniques	Scaling, rotation, and noise addition	CNN	Improved accuracy by 2% over non-augmented models
Rathod et al. (2020)	Deep learning with data augmentation	Color adjustment, zooming, and shifting	CNN	Enhanced model stability and a 5% increase in detection rate
Potato Leaf Disease Dataset (2025)	Deep learning-based approach	Extensive geometric and photometric transformations	CNN	Matched state-of-the-art accuracy, highlighting efficiency in processing large datasets

## 3 PotatoLeafNet: two-stage deep learning approach for accurate potato leaf disease detection and classification

**Figure 2:**
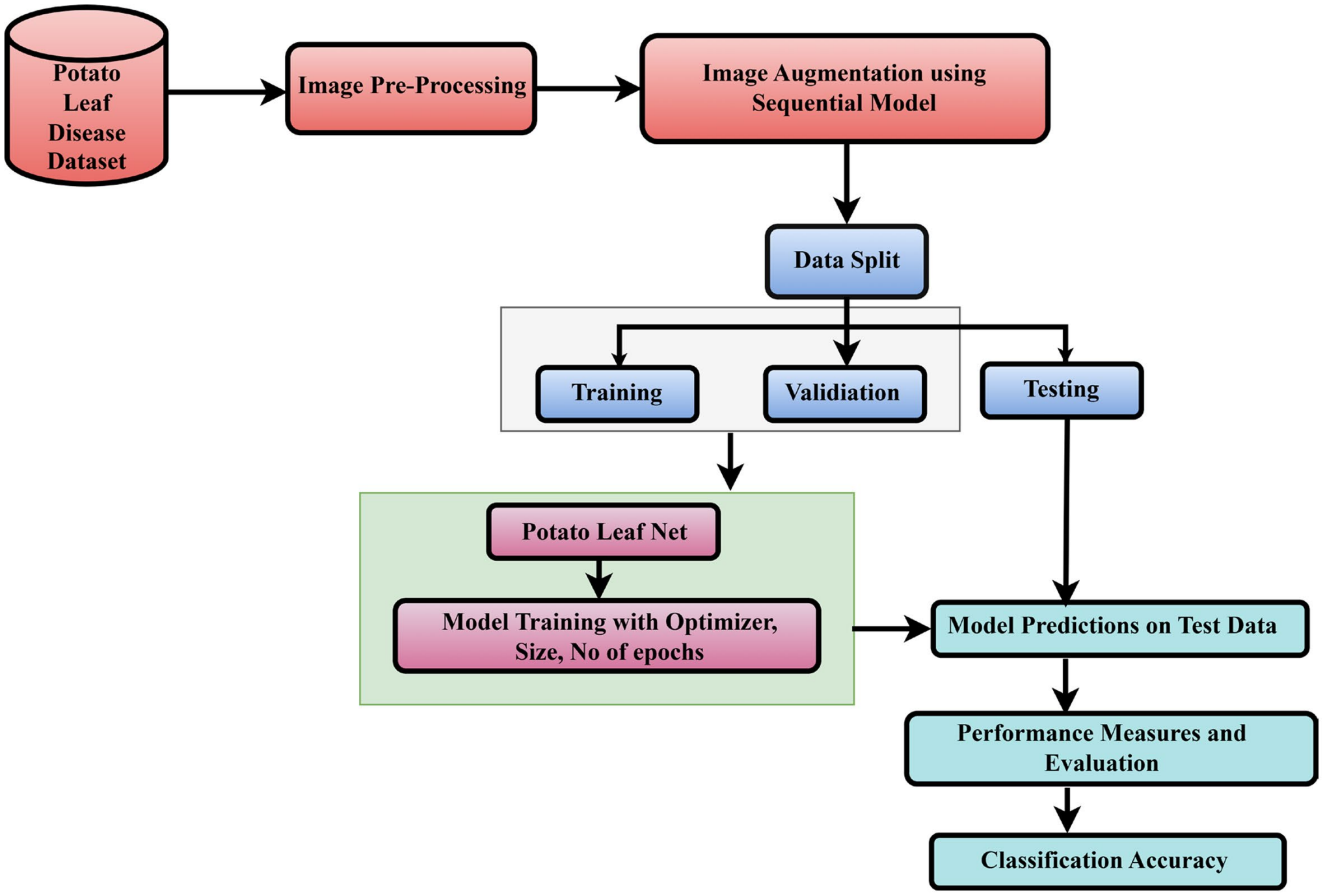
Flowchart for the proposed PotatoLeafNet two-stage CNN models for potato leaf disease detection and classification.

Challenges in deep learning approaches for potato leaf disease identification include inaccurate disease recognition, disease variations, high false rates, inadequate training samples, imbalanced classes, slow convergence, and improved accuracy. Deep learning methods have been extensively researched to identify and categorize potato leaf diseases. Early identification and treatment of potato leaves are crucial, but the lack of agricultural expertise in rural areas can be time-consuming and hindered. Acquiring such datasets remains a difficult task. Figure 2 represents the Flowchart for the proposed PotatoLeafNet two-stage CNN models for Potato Leaf Disease Detection and Classification.

### 3.1 Potato leaf disease dataset description

**Figure 3:**
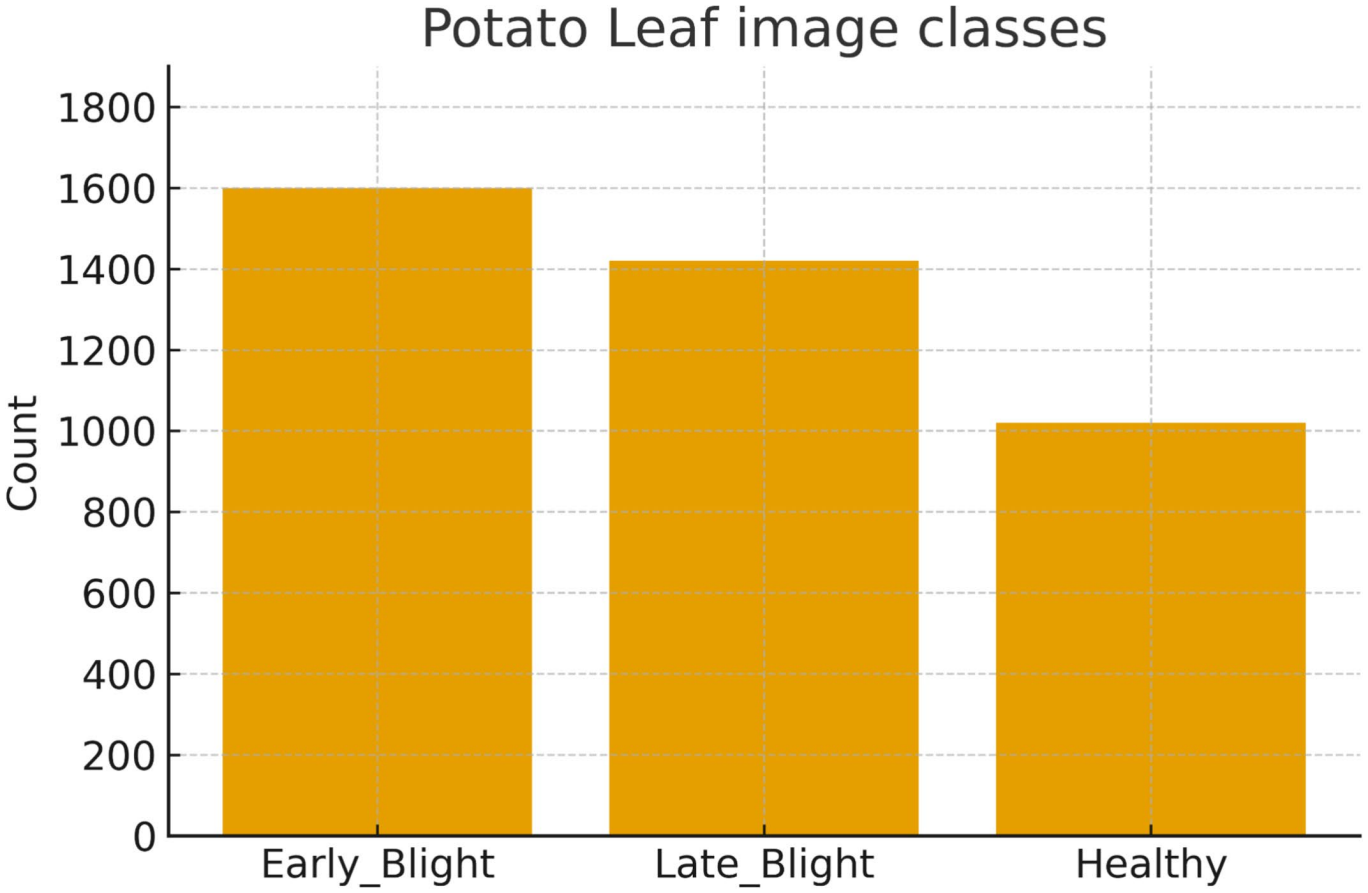
The potato leaf images from each of the 3 categories (original distribution).

Plant Village Dataset provides high-quality photos of different potato leaves (Mishra and Srivastava, 2019). Healthy, Early and Late Blight were photographed. Because of its availability, researchers have used the Plant Village dataset to simulate potato leaf diseases in the literature. This region-specific dataset includes few training and validation pictures and uneven class distribution. We need a fresh and comprehensive potato leaf dataset to address these research gaps. We curate the new dataset as the Potato Leaf Disease Dataset. Early Blight, containing 1,628 potato images, is the most critical disease affecting potatoes. The subsequent severe risk Late Blight contains 1,424 leaf images. We will examine 1,020 leaf images from the Healthy Next class for model training and testing. The dataset contains a complete 4,072 potato leaf images with three classes. The ratio between training, validation, and testing is 80:10:10. Figure 3 displays the potato leaf images from each of the three categories. Figure 3 presents the distribution of images across three classes of potato leaves: Early Blight, Late Blight, and Healthy. The Early Blight class has the largest number of images, just under 1,800, indicating a higher prevalence or focus on this category within the dataset. Late Blight follows closely, with a count near 1,600 images. The Healthy class has the fewest images, slightly above 1,400, suggesting a lesser representation in the dataset. This visual distribution highlights an imbalanced dataset which may be used for training a machine learning model to classify the health status of potato leaves.

### 3.2 Image processing and sequential image augmentation

Pre-processing was applied to all images to enhance lesion visibility, suppress background clutter, and standardize inputs prior to learning. Specifically, we performed contrast normalization to mitigate illumination variability, foreground–background separation to isolate the lamina, and spatial normalization to a common resolution. This stage improves the signal-to-noise ratio presented to the network and, in turn, the reliability of feature extraction for downstream classification (Hernandez-Valencia et al., 2020). To reduce storage and I/O overhead without compromising diagnostically salient content, we employed lossless and hybrid compression. Lossless codecs Huffman coding and run-length encoding (RLE) preserve the exact pixel values while exploiting redundancy to shrink file size (Yao et al., 2020). In the hybrid scheme, regions containing disease cues (lesion edges, texture) are preserved losslessly, whereas visually noncritical background is compressed lossily, striking a balance between fidelity and efficiency for large-scale training and deployment (TensorFlow Sequential Data Augmentation, 2025). (Compression is decoupled from resizing, it reduces bytes on disk/transfer, not spatial resolution.) leaf images captured in RGB are converted to grayscale (Gurucharan, 2020). Edge of Caution to recognize the edges in a leaf image and alleviate the irritation, unambiguous evidence is utilized (Powers, 2020). The external designs in leaf images are equal in how they are perceived from the edge. When the upper shape is taken as (p, q), the breadth and the level are (r, s), and these four centers do not settle the bobbing (Li et al., 2022). Each member of the upright hopping square is still a work in progress. The return on investment region is removed using the primary RGB leaf image’s coordinates (p + r, q + s). Finally, the dreaded leaf symbol may be put to rest.

#### 3.2.1 Sequential image augmentation

Sequential image augmentation can be incorporated into a sequential model in TensorFlow-Keras by using the “tf.keras.layers.experimental.pre-processing” module. This module provides various layers that can be added to the sequential model to apply different image augmentation techniques. These layers can be sequentially added to the model to apply random horizontal flipping, random rotation within a specific range, random zooming, and rescaling of pixel values. By adding the image augmentation pipeline as the first layer of the CNN sequential model, the subsequent layers can learn from augmented data, enabling the model to generalize better and handle variations in the input images.

The ‘image_augmentation’ sequential model used in this research represents an augmentation pipeline using various pre-processing layers.

- **Random Rotation**: This layer randomly rotates the input image by a maximum of 0.2 radians (approximately 11.5 degrees) in a counterclockwise direction. It introduces variability by simulating different object orientations in the image.
- **Random Zoom**: This layer applies random zooming to the input image, ranging from 0 to 20% of the original size. It helps capture different scales or perspectives of the object.
- **Random Height**: This layer randomly changes the height of the input image by scaling it between 80 and 120% of the original height. It adds variability by modifying the image’s aspect ratio.
- **Random Width**: This layer randomly changes the width of the input image by scaling it between 80 and 120% of the original width. Like ‘Random Height’ it introduces variability by modifying the aspect ratio.
- **Rescaling:** This layer rescales the pixel values of the input image to a range of [0, 1]. In this case, it divides each pixel value by 255, assuming the input image has an 8-bit color depth. Rescaling is a typical pre-processing step to ensure numerical stability and convergence during model training.

The ‘image_augmentation’ model can be used as a pre-processing step in your overall CNN model pipeline. It applies random transformations to the input images during training, enhancing the diversity and robustness of the data.

### 3.3 Augmented data split into training, validation, and testing

The potato leaf disease dataset was divided into training, validation, and testing sets using 80, 10, and 10% split ratios. Sequential image augmentation procedures on the training set reduced overfitting and increased dataset variation. Rescaling, rotating, modifying shear and zoom ranges, flipping horizontally, adjusting brightness, and moving channels were these tactics. CNN model predictions were improved using Adam optimization with forward and backpropagation. Thus, CNN model output accuracy was ensured. The validation and testing sets contained 20% of the training set, which included images of early, robust, and late blight. The PotatoLeafNet model categorized practice pictures and predicted class labels on the training dataset.

### 3.4 PotatoLeafNet—potato leaf-based CNN for potato leaf disease detection and classification

The existing literature on deep learning approaches reveals several challenges, including misdiagnosis of potato leaf identification, variations in potato leaves due to different varieties, and environmental factors. Early detection and management of potato diseases are crucial, but the process is time-consuming, and access to agricultural expertise is limited in rural areas (Alzakari et al., 2025). CNNs have shown remarkable progress in image-based recognition, eliminating the need for extensive image pre-processing and enabling automatic feature selection (Weng et al., 2024). However, the availability of large datasets specifically for potato leaf challenges remains a significant obstacle.

#### 3.4.1 Convolutional neural network (CNN) model

CNNs were developed to process the data represented in grid-like structures like images. The pixels in an image are arranged in a grid, and the value of each pixel determines its hue and luminance. Likewise, each neuron in a CNN processes information within its receptive field. Like how the human brain processes visual information, CNN layers detect simpler patterns first, then more complex ones as the layer progresses.

**Figure 4:**
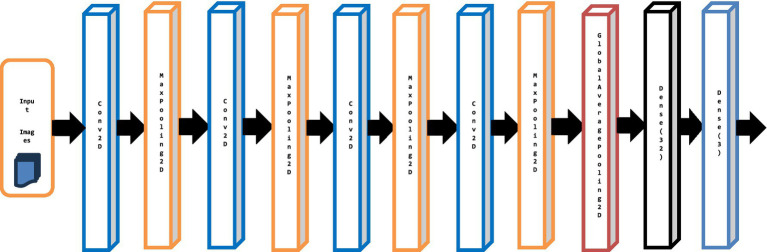
Architecture of the proposed PotatoLeafNet model for potato leaf detection and classification.

Convolutional neural networks have input, hidden, and output layers. Convolution, normalization, pooling, and fully-connected layers lie between the output and input layers. The convolutional layer’s filters create classification feature maps. Image processing uses ReLU. This paper proposes an improved fine-grained robust PotatoLeafNet model for classifying potato leaf diseases. To minimize the size of the leaf picture and create several images, image pre-processing and sequential image augmentation methods are utilized at the first level. A CNN learning model using a CNN has been established at the next level to identify sick leaves in the images. The PotatoLeafNet model for potato leaf disease prediction is shown in Figure 4.

CNN models have numerous convolutional, pooling, and fully linked layers. Because of its high complexity, a neural network can develop hierarchical representations of the input data, which are crucial for precise categorization. Section 3.6 presents a detailed pseudo code for the proposed CNN model for Potato Leaf detection and classification. The Convolutional Neural Network model consists of multiple layers, including Conv2D, Batch Normalization, Max Pooling, and Activation functions. Here is a description of the model architecture:

1. **Conv2D layers**: The convolutional operation distinguishes a CNN from other neural networks. The basic form of convolution consists of two functions that take real numbers as arguments. To explain convolution, we can pretend that it is possible to track where a car is using a laser that gives an output: *x(t)*, where x is the car’s position in time step *t*. Several measurements can be taken to reduce possible noise during the measurements, and an average value of them is used as the measurement value. Later measurements have greater value than the older ones. Therefore a weight function, w(a), is used, where a represents how old a measurement is. The weight function w must be a valid density function. If these weighted average measurements are performed every time step, it can be described with a function, s known as the Convolution function.
s(t)=∫x(a)w(t−a)da (1)

In CNN terminology, the first argument in the convolution function is called the input, and the second is called the kernel; what is returned is called the *feature map*.


s(t)=(x∗w)(t) (2)

For the example with the car above to be realistic, the data cannot be collected in each time step when the amount had become too large, but in regular intervals, for example, every second or minute. In such a case, the time variable t would only be of integer type; likewise, the variables x and w, then the mathematical discrete convolution, can be defined as.


$$s(t)=(x*w)(t)=\sum_{a=\infty }^{\infty }x(a)w\;(t-a)$$ (3)

The model includes 5 Conv2D layers. Conv2D performs convolution operations on the input image to extract features. Each Conv2D layer consists of a set of learnable filters that scan the input image and produce feature maps. These filters capture different patterns and features at different scales.

1. **Batch Normalization layers**: The batch normalized activation is
$${\underline{x}}_{i}=\frac{x_{i}-\mu_{B}}{\sqrt{\sigma_{B}^{2}+e}}$$ (4)

Where $\mu_{B}=\frac{1}{m}\sum_{i=1}^{m}\mathrm{xi}$ is the batch mean, and $\sigma^{2}B=\frac{1}{m}\sum_{i=1}^{m}{\left(\mathrm{xi}-\mathrm{\mu B}\right)}^{2}$ is the batch variance. Batch Normalization is applied after each Conv2D layer. Adjusting the mean and variance helps normalize the previous layer’s output. It helps stabilize the training process and improve the model’s overall performance.

1. **Max Pooling layers**: Max Pooling precedes each Conv2D layer. The maximum value inside a pool size lower feature map spatial dimension. Max Pooling helps down-sampling the feature maps and extracting the most essential features while reducing computational complexity.
$$h_{\mathrm{xy}}^{1}=\max_{i=0\ldots .s,j=0\ldots ..s}\;h^{1}\;(x+i)(y+i)$$ (5)
2. **Activation functions**: 7 activation functions are used in the model’s layers. Activation functions allow the model to learn complicated patterns and make nonlinear judgments. CNNs often use ReLU, sigmoid, and tanh activation functions to feed one layer’s output into the next.
$$\text{ReLU}(x_{i})=(0,x_{i})\;$$ (6)

Combining Conv2D layers, Batch Normalization, Max Pooling, and Activation functions helps the CNN model extract and learn intricate features from the input data effectively. It allows the model to capture the information for accurate classification or detection tasks. The model uses convolutional operations, sequential image augmentation, and global average pooling to accurately and efficiently classify potato leaf diseases. Table 2 shows the proposed PotatoLeafNet model architecture summary, and Figure 4 represents the proposed PotatoLeafNet model for Potato Leaf detection and classification.

- **Sequential Image Augmentation**: The input images are enhanced by this layer using various image augmentation methods, including random flipping, rotation, zooming, and rescaling. It transforms the pictures to prepare them for better generalization.
- **Conv2D (60 filters, 3 × 3, ReLU):** This convolutional layer performs convolutions on the input images using 60 filters of size 3 × 3 and applies the ReLU activation function. It extracts 60 different features from the input images, resulting in an output shape of (None, 222, 222, 60).
- **MaxPooling2D (pool size: 2 × 2):** By taking the highest value inside each 2 × 2 region, this max pooling layer decreases the spatial dimensions of the feature maps by a factor of 2 (Tiwari et al., 2020). It helps in reducing the spatial dimensions and capturing the most salient features, resulting in an output shape of (None, 111, 111, 60).
- **GlobalAveragePooling2D**: This layer performs global average pooling, reducing the spatial dimensions to a single value per channel. It summarizes spatial information and retains essential features. Resulting in an output shape of (None, 60).
- **Dense (128 units, ReLU):** This fully connected dense layer with 128 units applies the ReLU activation function. It introduces non-linearity and learns high-level representations based on the extracted features from previous layers. Resulting in an output shape of (None, 128).
- **Dense (total_classes units, Softmax):** The softmax activation function is used in the last dense layer, which has units equal to the entire number of classes in the classification job. It generates class probabilities, which show the chance that each input picture belongs to a specific class of illness.

**Table 2:** Summary of the proposed PotatoLeafNet model architecture.

Layer	Output shape	Number of parameters	Unique Configuration
Sequential Image Augmentation	(None, 224, 224, 3)	0	Custom augmentation settings for potato leaf images
Conv2D (60 filters, 3 × 3, ReLU)	(None, 222, 222, 60)	1,740	Optimized for initial feature extraction
MaxPooling2D (pool size: 2×2)	(None, 111, 111, 60)	0	Reduces dimensionality, retains critical spatial features
Conv2D (60 filters, 3 × 3, ReLU)	(None, 109, 109, 60)	32,460	Additional depth to capture complex features
MaxPooling2D (pool size: 2×2)	(None, 54, 54, 60)	0	Further reduces spatial dimensions, focuses on feature pooling
Conv2D (60 filters, 3 × 3, ReLU)	(None, 52, 52, 60)	32,460	Increases model’s capacity to learn detailed features
MaxPooling2D (pool size: 2×2)	(None, 26, 26, 60)	0	Enhances the abstraction level of the features
Conv2D (60 filters, 3 × 3, ReLU)	(None, 24, 24, 60)	32,460	Prepares for high-level reasoning by the network
MaxPooling2D (pool size: 2×2)	(None, 12, 12, 60)	0	Last pooling step to compact features before classification
GlobalAveragePooling2D	(None, 60)	0	Reduces each feature map to a single number to minimize overfitting
Dense (128 units, ReLU)	(None, 128)	7,808	Dense layer to combine features into higher-level attributes
Dense (total_classes units, Softmax)	(None, total_classes)	total_classes	Tailored for the specific number of disease classes

These layers form the PotatoLeafNet model, which combines sequential image augmentation, convolutional layers for feature extraction, pooling layers for spatial dimension reduction, global average pooling for summarization, and fully connected layers for classification. The model is trained to classify potato leaf disease images into their respective classes.

### 3.5 Performance measure

Multiple metrics are used to evaluate the success of a network. Using different task metrics helps represent the network’s ability to solve a given problem. The evaluation metrics can use true positive (TP), false positive (FP), true negative (TN), and false negative (FN).

**Classification Accuracy:** is determined by the ratio of correct prediction to total predictions.


$$\text{Accu}r\mathrm{acy}=\frac{\text{Number of Correct Predictions}}{\text{Total number of Predictions}}$$ (7)

**Precision:** Precision determines with what precision the network places images in the positive category. Precision is calculated as follows:


$$\text{Precision}=\frac{\mathrm{TP}}{\mathrm{TP}+\mathrm{FP}}$$ (8)

**Recall:** Recall indicates how many positive images the network recorded. The recall is calculated as follows:


$$\text{Recall}\;\frac{\mathrm{TP}}{\mathrm{TP}+\mathrm{FN}}$$ (9)

**F1-Score**: F1-Score is a combination of Precision and Recall. The calculation is as follows:


$$F1-\text{Score}=2*\frac{\text{Precision}*\text{Recall}}{\text{Precision}+\text{Recall}}$$ (10)

### 3.6 Algorithm of the proposed PotatoLeafNet model for potato leaf detection and classification

PotatoLeafNet model for potato leaf detection and classification shown in Algorithm 1.

ALGORITHM 1 **Input: Potato Leaf Disease Dataset**
**Output: Disease Detection and Classification of Potato LeavesStep1:** Acquire the Potato images with Late Blight, Early Blight, and Healthy
**Step2:** Loading the data (X_train,y_train), (X_test,y_test)=image.load_data()
**Step 3:** first stage of PotatoLeafNet for sequential image augmentation model for image augmentation with 6 layers. Each layer is performing RandomFlip, RandomRotation(0.2), RandomZoom(0.2), Rescaling(1./255)
**Step 4**: Put the correct labels on the pictures of the potato leaf images.
**Step 5**: Sort photos into categories using the available class labels from the training and testing datasets.
**Step 6:** Initialize the parameters image size, epochs, batch size, and train and test image labels.
**Step 7:** The Second stage of the PotatoLeafNet Model uses 4 blocks containing Conv2D, Max Pool2D, and GloalAveragePooling2D, followed by Desne layers. The total number of layers is 11.
**Step 8:** Evaluate the trained model using a separate testing dataset.
   Calculate the test loss and accuracy of the model.
**Step 9:** Check the accuracy of the proposed models, and see how they stack up against the rest of the CNN models out there. Make predictions on new data
predictions = model.predict(new_images).

## 4 Results and discussion

All experiments were implemented in Python using TensorFlow and Keras, optimizing a categorical cross-entropy objective with Adam and a learning-rate schedule; runs were executed on a server equipped with an NVIDIA P100 GPU, an Intel i5 CPU, and 8 GB RAM. The evaluation centered on four aims: reliably tri-classifying potato leaf images into Early Blight, Late Blight, and Healthy categories, quantifying the effect of a fixed sequential image-augmentation pipeline during training on the PotatoLeafNet’s performance; benchmarking PotatoLeafNet against contemporary convolutional baselines; and situating the empirical findings within prior deep-learning studies on potato leaf disease identification.

### 4.1 Datasets description

We curated a diverse, high-quality corpus of potato leaf images spanning Healthy, Early Blight, and Late Blight classes. Training uses the PlantVillage Potato subset, a widely used, fully open benchmark for potato leaf disease recognition, to mitigate its limitations and class imbalance, we additionally compiled a complementary Potato Leaf Disease Dataset with 4,072 images 1,628 Early Blight, 1,424 Late Blight, and 1,020 Healthy. To assess real-world generalization beyond PlantVillage, we conduct cross-dataset validation: models trained on PlantVillage are evaluated, without further tuning, on PlantDoc (Singh et al., 2020) (in-situ scenes with variable lighting, occlusion, and background clutter) and on Shabrina et al. (2023) field collection (uncontrolled conditions; seven potato classes remapped to {Healthy, Early Blight, Late Blight} for comparability). We report Accuracy, Macro-F1, per-class Precision/Recall, Matthews Correlation Coefficient (MCC), and Expected Calibration Error (ECE), and provide confusion matrices and Grad-CAM overlays. Finally, a few-shot field-adaptation ablation (10% labeled field images) quantifies domain shift and the benefit of lightweight adaptation.

### 4.2 Data pre-processing and sequential image augmentation

**Figure 5:**
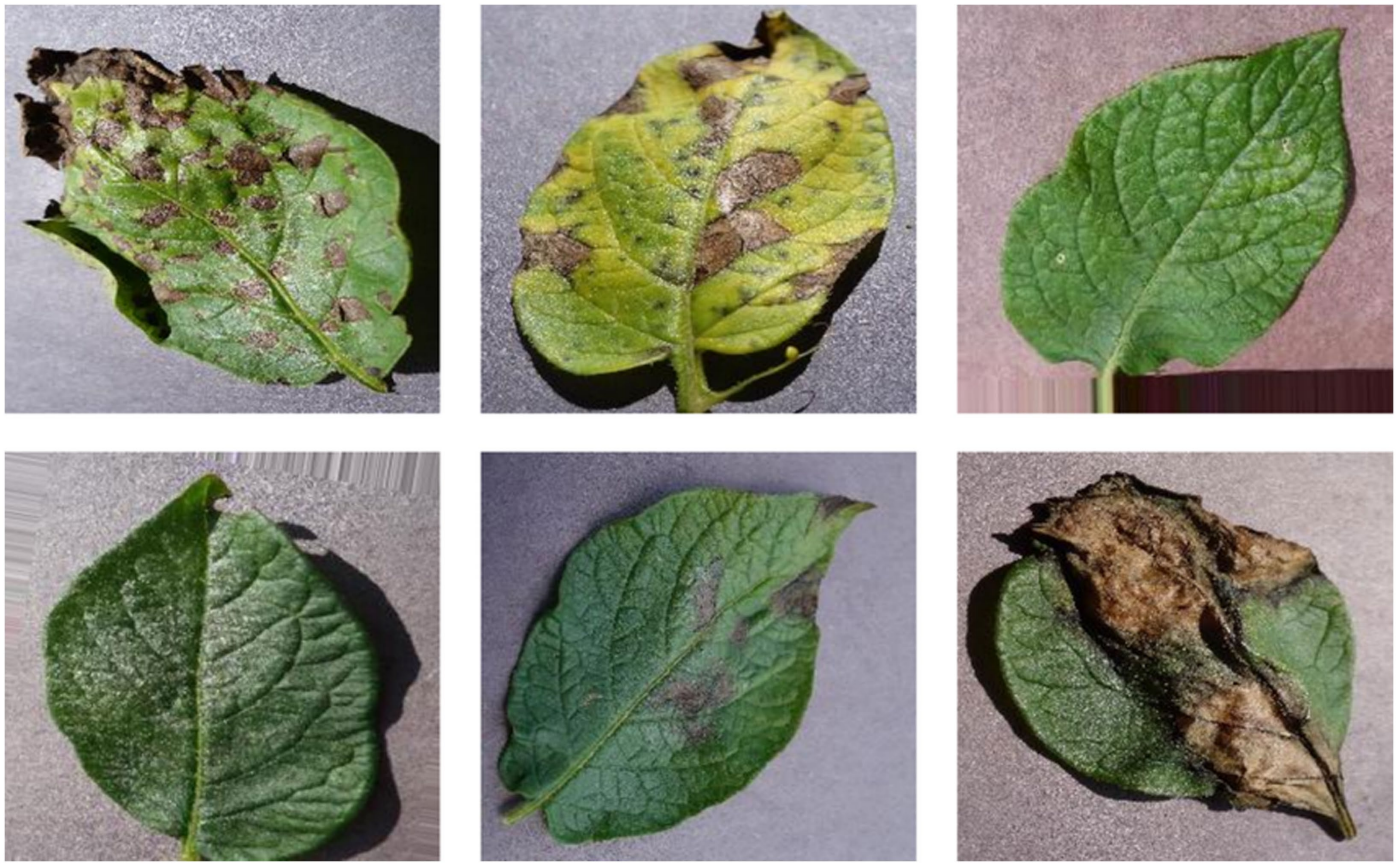
The pre-processed images with a resolution of 224×224.

All images were prepared for CNN training by converting them to RGB float tensors and resizing uniformly to 224 × 224 pixels. Pixel intensities were normalized to [0,1] to stabilize optimization. The dataset comprised 4,072 potato-leaf images across three classes Healthy, Early Blight, and Late Blight. To enhance the robustness and generalizability of PotatoLeafNet, we applied a fixed-order (sequential) image-augmentation pipeline in Keras on the training split only, thereby increasing appearance diversity while preserving label integrity and class balance. The augmentation sequence consisted of rotation (±25°), width shift (±0.10), height shift (±0.10), shear (0.20), random zoom (up to 0.20), horizontal flip, brightness jitter (0.5–1.0), and channel shift (0.05). Applying this policy expanded the training corpus to 6,000 images, balanced as 2,000 per class, which mitigated class imbalance and improved generalization across diverse disease manifestations. Figure 5 illustrates representative pre-processed images at the target 224 × 224 resolution.

#### 4.2.1 Evaluation protocol

We access robustness with Repeated K-Stratified K-Fold (*k* = 5, *r* = 3; total *N* = 15 fits). For fold f, models are trained on $D_{f}^{\text{train}}$ , validated on $D_{f}^{\mathrm{val}}$ (early stopping, best checkpoint), and evaluated on $D_{f}^{\text{test}}$ . We report Accuracy, Macro-precision, Macro- Recall, Macro-F1 as (μ±σ) over all N runs, with 95% cls via the t-distribution:


$$\begin{array}{l} \mu =\frac{1}{N\;}\;\sum_{i=1}^{N}m_{i},\sigma =\frac{1}{N-1}\sum_{i=1}^{N}{\left(m_{i}-\mu \right)}^{2},\\ CI_{95\%}=\mu \pm t_{0.975,N-1}\frac{\sigma }{\surd n} \end{array}$$ (11)

For pairwise model comparisons on identical folds we apply paired t-tests and Wilcoxon signed-rank tests, prediction-level differences are examined with the McNemar test. We report Hedges’ g and cliff’s δ as effect sizes and apply Holm-Bonferroni to control family-wise error. To quantify optimization stochasticity, we additionally train each model on the canonical split with 5 distinct random seeds and report mean ± SD Protocol details Input Size 224*2,224, identical preprocessing/normalization across models, no augmentation on validation/test, Stratification by class (Healthy, Early Blight, Late Blight), fixed fold indices shared by all models, deterministic settings (global seed, seeded data loaders, CuDNN deterministic).

### 4.3 PotatoLeafNet performance on potato leaf disease dataset

**Figure 6:**
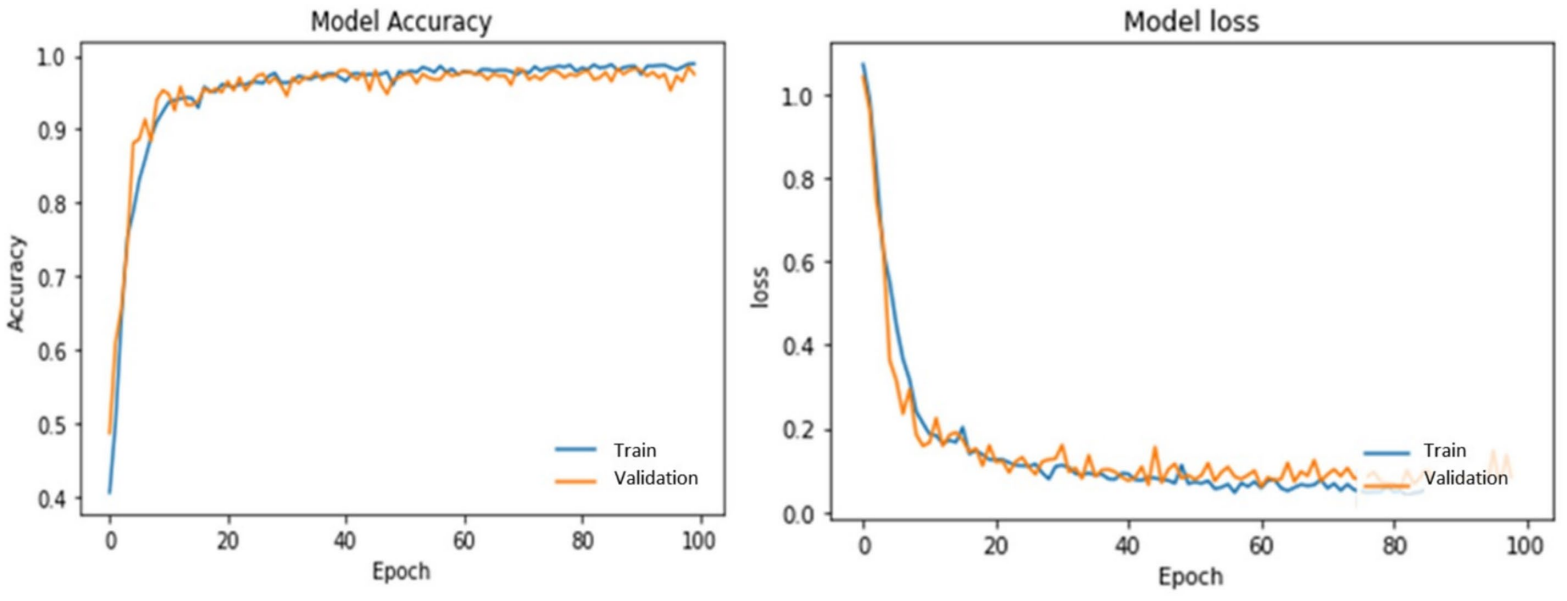
The performance of PotatoLeafNet on potato leaf disease with sequential image augmentation for 100 epochs.

**Figure 7:**
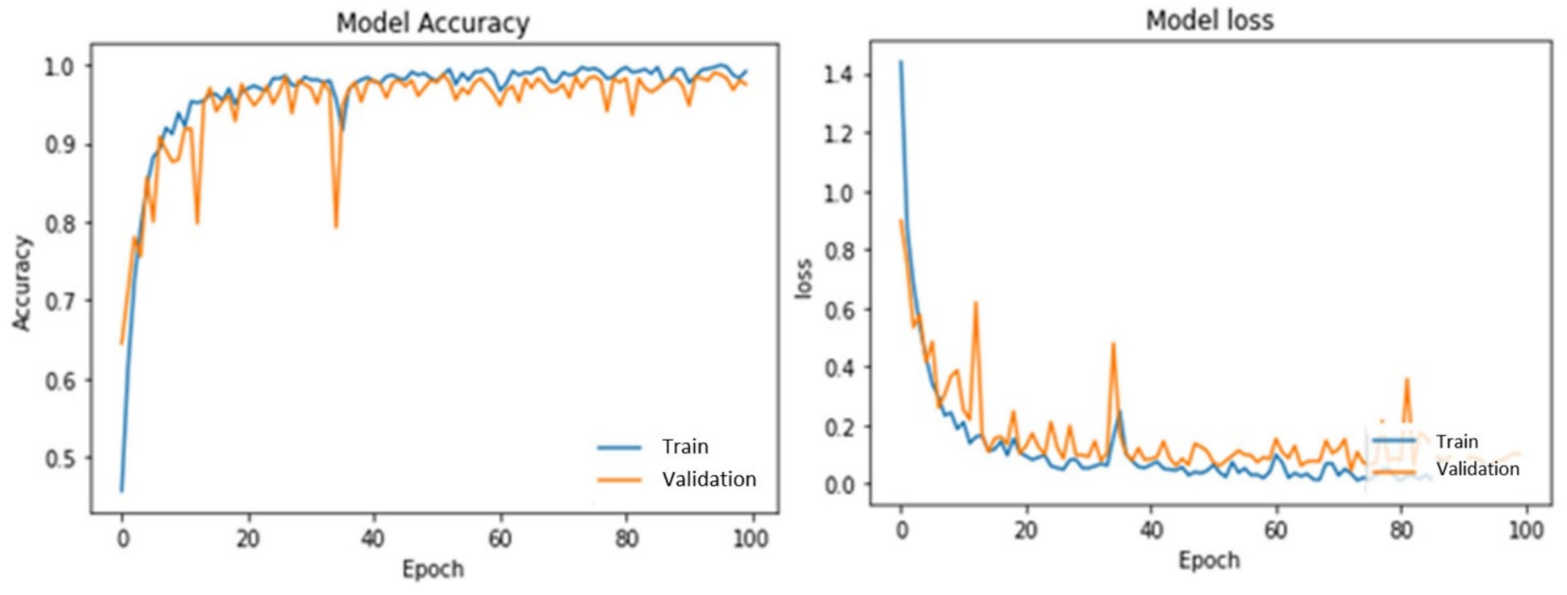
Performance of PotatoLeafNet on potato leaf disease without sequential image augmentation for 100 epochs.

**Figure 8:**
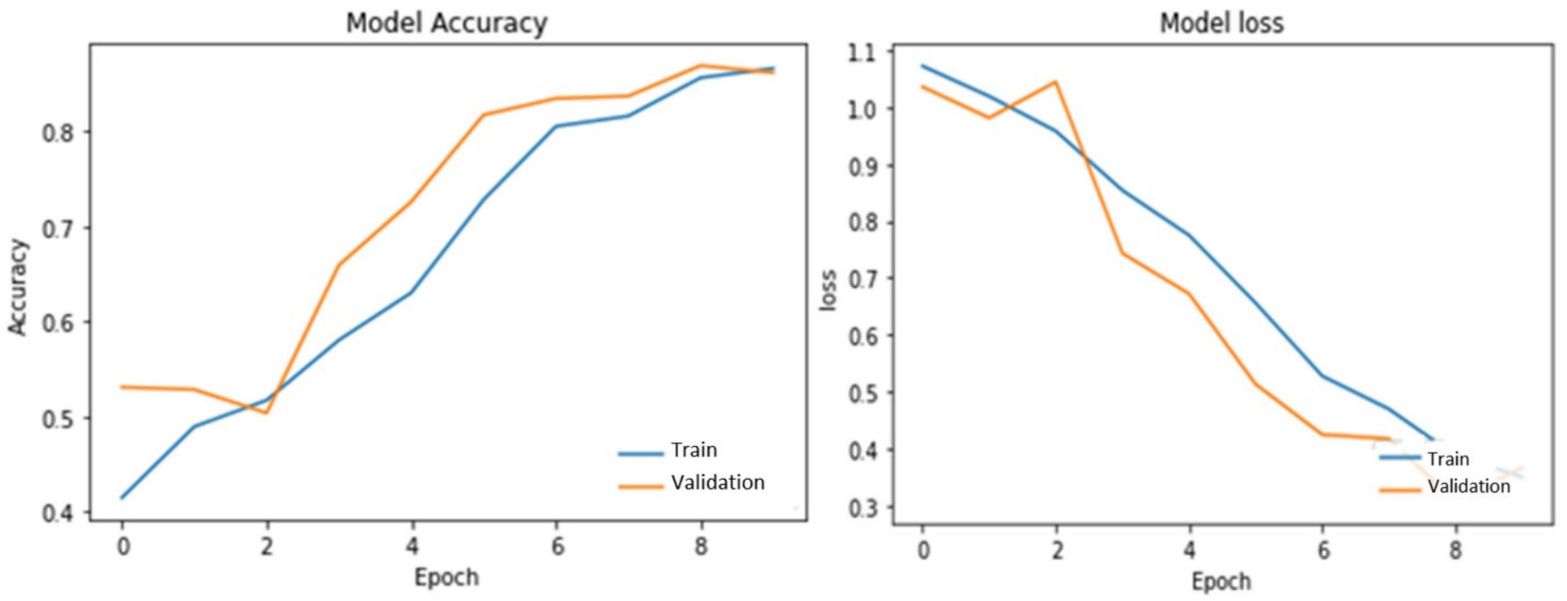
Performance of PotatoLeafNet on potato leaf disease with sequential image augmentation for 10 epochs.

**Figure 9:**
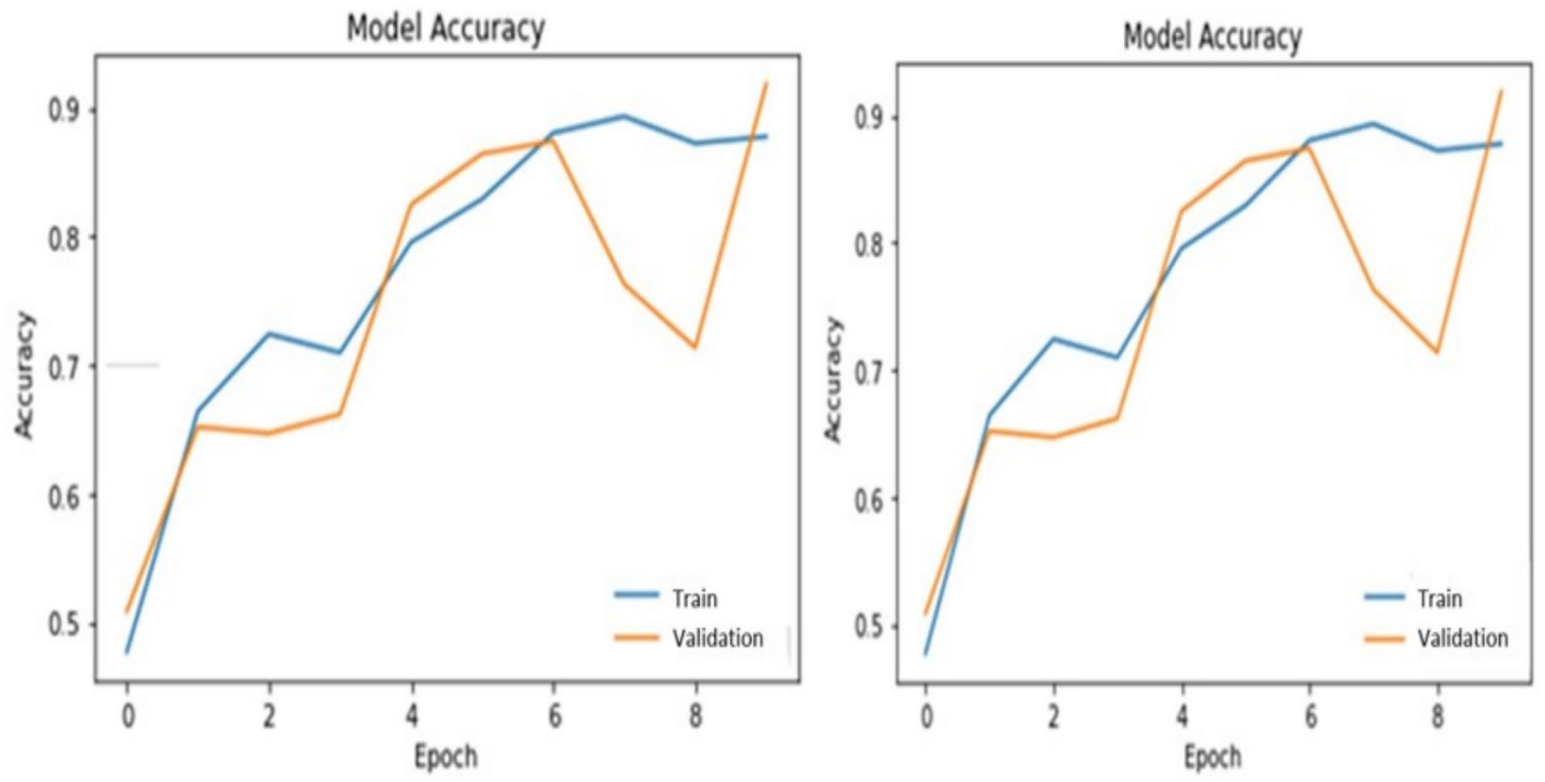
Performance of PotatoLeafNet on potato leaf disease without sequential image augmentation for 10 epochs.

Table 3 illustrates PotatoLeafNet configurations. The dataset shows the PotatoLeafNet model, which changes internal parameters to improve performance during training. The model learns to extract important traits and properly characterize Late, early blight, and Healthy over several epochs. Each epoch’s accuracy and loss statistics show model performance. Accuracy is the percentage of correctly predicted instances concerning actual. Figures 6–9 demonstrate the accuracy of PotatoLeafNet architectures’ potato leaf disease detection and classification.

**Table 3:** Parameters used in the PotatoLeafNet model.

S. No	Parameter used	Value
1	Training epochs	10 and 100
2	Optimizer	Adam
3	Batch size	32
4	Drop out	0.25
5	Image size	224 × 224
6	Kernel size	3
7	Data shuffle	True for every 1,000 images
8	No of classes	3
9	Callback	True on model checkpoint
10	Loss function	Cross entropy
11	Learning rate	0.001

The two-stage CNN model evaluates PotatoLeafNet on potato leaf disease data. Potato leaf disease datasets employ six-layer sequential image enhancement. A freshly developed and fine-tuned CNN model analyzes the training dataset’s accuracy. The PotatoLeafNet model was optimized with sequential picture augmentation. PotatoLeafNet model training requires a huge sample.

Figure 6 summarizes the learning dynamics of PotatoLeafNet trained for 100 epochs with sequential image augmentation on the potato leaf disease dataset. The training accuracy rapidly increases and saturates at 98.92% with a final training loss of 0.0356, while the validation accuracy stabilizes around 97.53% with a closely aligned validation loss curve. The small gap between training and validation accuracies, together with the monotonically decreasing and non-diverging loss trajectories, indicates that the augmented model generalizes well beyond the training set. On the held-out test set, PotatoLeafNet attains 98.52% accuracy, confirming that sequential image augmentation provides effective regularization and supports highly reliable classification of healthy, early blight, and late blight leaves.

Figure 7 shows the behavior of a single-stage PotatoLeafNet trained for 100 epochs without sequential image augmentation. In this setting, the model reaches 96.17% training accuracy with a training loss of 0.2250 and achieves 96.52% validation accuracy, but the validation accuracy and loss curves exhibit noticeably larger oscillations than in Figure 6. Test accuracy is reduced to 96.01%, i.e., 2.51 percentage points below the augmented two-stage PotatoLeafNet, and the substantially higher training loss further reflects less stable optimization. Comparing Figures 6, 7 demonstrates that sequential image augmentation not only increases training/validation/test accuracy by +2.75/+1.01/+2.51 points, respectively, but also yields smoother validation trajectories and lower loss, highlighting its role as an effective regularizer.

Figure 8 depicts PotatoLeafNet trained for only 10 epochs with sequential image augmentation. Even under this short training regime, the model already reaches 88.22% training accuracy with a training loss of 0.3535, while the validation accuracy rises to 86.91% and the validation loss decreases steadily. The corresponding test accuracy of 88.15% confirms that the augmented model generalizes well even before full convergence. These dynamics indicate that augmentation quickly exposes the network to diverse views of each class, enabling the model to acquire discriminative features early in training and to maintain a small and stable train–validation gap.

Figure 9 presents the same 10-epoch training schedule without sequential image augmentation. In this baseline configuration, the model attains 87.82% training accuracy and 0.3410 training loss, with validation and test accuracies of 85.82 and 86.91%, respectively. Compared with Figure 8, both validation and test accuracies are consistently lower and the gap between training and validation curves is slightly larger, suggesting mild overfitting when the network is trained on a less diverse set of images. The corresponding loss curve also shows a less smooth descent, pointing to reduced robustness of the optimization process. Together, Figures 8, 9 illustrate that, even at an early training stage, sequential image augmentation improves generalization and stabilizes the learning dynamics of PotatoLeafNet.

#### 4.3.1 Model efficiency and parameters

To contextualize deployment cost alongside accuracy (Table 4), we benchmarked five models under a unified protocol and report tuning strategy, wall-clock training time, parameter count, and FP32 memory footprint (4 bytes per parameter). The proposed PotatoLeafNet used manual tuning with ReduceLROnPlateau and ModelCheckpoint, a fixed sequential augmentation policy, and Adam (lr = 1e-3); it trained in 1.2 h, contains 16.5 M parameters, and occupies 66 MB of memory (moderate complexity). The ResNet-50 + VGG-16 fusion, using transfer learning with fine-tuning, trained in 1.5 h and comprises 164.00 M parameters (656 MB) [or 38.30 M, 153 MB, if reported without the ImageNet classifier]; VGG-16 + MobileNetV2 with grid-search tuning trained in 1.8 h and totals 141.90 M parameters (567.6 MB) [or 16.97 M, 67.9 MB, without top]. MobileNetV2, tuned via random search, trained in 0.8 h, has 3.54 M parameters (14 MB) and low complexity. Inception-V3, fine-tuned via standard transfer learning, trained in 1.2 h and includes 23.85 M parameters (≈95 MB) with moderate complexity. These results show that PotatoLeafNet is far smaller than fusion baselines and within an order of magnitude of MobileNetV2; post-training INT8 quantization typically reduces memory by 4 × (e.g., PotatoLeafNet to 16.5 MB, MobileNetV2 to 3.5 MB), improving feasibility for real-time mobile/web deployment.

**Table 4:** Comparison of model efficiency and parameter complexity.

Model	Parameter tuning method	Training time (h)	Parameter count (Millions)	Hyperparameter complexity
Proposed PotatoLeafNet	Manual tuning with ReduceLROnPlateau + ModelCheckpoint; fixed sequential augmentation; Adam (lr = 1e-3)	1.2	16.5 M	Moderate
ResNet-50 + VGG-16	Transfer Learning + Fine-Tuning	1.5	138.36 M	High
VGG-16 + MobileNetV2	Transfer Learning + Grid Search	1.8	141.67 M	High
MobileNetV2	Random Search	0.8	3.54 M	Low
Inception-V3	Neural Architecture Search (NAS)	1.2	23.85 M	Moderate

### 4.4 Comparison of accuracy between proposed method and existing studies

Table 5 benchmarks the proposed PotatoLeafNet against recent potato-disease studies spanning handcrafted descriptors with classical classifiers (Ala’a), transfer-learned CNNs (Nur et al., 2025; Shah et al., 2025), compact bespoke CNNs (Kaur et al., 2025; Salihu et al., 2025), hybrid CNN–Transformer designs (Sinamenye et al., 2025; Zhang et al., 2025), and a non-image tabular risk-forecasting approach (Radwan et al., 2025). Despite heterogeneity in data sources and class definitions, PotatoLeafNet attains 98.52% accuracy on PlantVillage (Healthy/Early/Late), placing it among the top performers while using a compact 11-layer 3 × 3 convolutional stack and a fixed sequential photometric augmentation policy. Notably, several comparators optimize for different modalities (e.g., meteorological risk factors) or field-like imagery; therefore, results are indicative rather than strictly commensurate, and cross-dataset validation remains essential for assessing real-world robustness.

**Table 5:** Comparison analysis of PotatoLeafNet with existing studies on potato leaf disease dataset.

Study [Ref]	Year	Model/approach	Dataset (classes)	Reported accuracy (%)	Core techniques/notes
Radwan et al. (2025)	2025	MLP with binary Greylag Goose Optimization for feature selection, Compared with LR, SVM, KNN, Gradient Boosting	4,000 meteorological records for early and late blight risk	98.30	K-means, PCA, copula analysis for structure, tabular risk forecasting complementary to image-based screening
Chen and Liu (2025)	2025	CBSNet with Channel Reconstruction Multi-Scale convolution Spatial Triple Attention, Bat-Lion strategy	Self-built photo leaf set (Healthy, Early blight, Late blight)	92.04	Target tiny lesions, blurred edges, and noise, attention driven multi-scale feature extraction
Sinamenye et al. (2025)	2025	Hybrid EfficientNetV2-B3 with vision Transformer	Potato Leaf Disease Dataset (field-like variability)	85.06	Combines local convolutional features and global transformer context for generalization
Salihu et al. (2025)	2025	CNN trained with Adam	Curated set (Healthy, Early blight, Late blight)	96.88	Scaling augmentation, normalization, confusion-matrix based evaluation
Ala’a (2025)	2025	Generalized Jones polynomial features with SVM Classifier	Plant Village (Potato)	98.45	Pipeline: Preprocessing, GJP feature extraction, dimensionality reduction, SVM, Strong handcrafted descriptor baseline
Zhang et al. (2025)	2025	VGG16S (GAP with CBAM and Leaky ReLU, about 15 M parameters)	Early blight and viral disease set with augmentation	97.87	Response-Surface hyperparameter tuning, ablations and public dataset tests reported
Kaur et al. (2025)	2025	PotConvNet (Compact CNN)	Two Potato datasets with defined splits	97.78	Resizing, normalization, augmentation, high accuracy on Dataset 1 and Strong cross-dataset results
Nur et al. (2025)	2025	Inceptionv3 with transfer learning and targeted fine tunning	Domain-Specific potato leaf set	97.78	Fine-tuned terminal layers, efficient and practical
Shah et al. (2025)	2025	PLDC-Net with EfficientNet-B1 backbone and SVM Classifier	Balanced multi-disease image set from online sources	98.39	Emphasizes data balancing and robust augmentation, evaluated on unseen images
Proposed model	2025	PotatoLeafNet	Plant Village (Potato)	98.52	Photometric transforms applied before learning; same policy across classes.11-layer convolutional stack with 3 × 3 kernels (PotatoLeafNet)

### 4.5 Comparative performance

Under the same training–evaluation protocol, the Proposed model achieves 98.52% accuracy, 98.67% precision, 99.67% recall, 99.16% F1-score, and 1.00 AUC. Relative to ResNet-50 + VGG-16 (97.10, 95.00, 94.00, 94.00%, 0.98), this corresponds to absolute gains of +1.42 pp. accuracy, +3.67 pp. precision, +5.67 pp. recall, +5.16 pp F1, and +0.02 AUC Table 6. Against VGG-16 + MobileNetV2 (94.80, 92.00, 91.00, 91.00%, 0.93), the gains are +3.72 pp, +6.67 pp, +8.67 pp, +8.16 pp, and +0.07. Versus MobileNetV2 (93.20, 91.00, 90.00, 90.00%, 0.92), the improvements are +5.32 pp, +7.67 pp, +9.67 pp, +9.16 pp, and +0.08; and versus Inception-V3 (92.50, 90.00, 89.00, 89.00%, 0.91), they are +6.02 pp, +8.67 pp, +10.67 pp, +10.16 pp, and +0.09. The consistent, largest margin in recall indicates the Proposed model substantially reduces false negatives critical for early disease screening while simultaneously delivering the best precision, F1, and AUC among all baselines.

**Table 6:** Comparative performance of the proposed model and deep learning.

Model	Accuracy (%)	Precision (%)	Recall (%)	F1-score (%)	AUC
Proposed model	98.52	98.67	99.67	99.16	1.00
ResNet-50 + VGG-16	97.1	0.95	0.94	0.94	0.98
VGG-16 + MobileNetV2	94.8	0.92	0.91	0.91	0.93
MobileNetV2	93.2	0.91	0.90	0.90	0.92
Inception-V3	92.5	0.90	0.89	0.89	0.91

### 4.6 PotatoLeafNet model performance on correctly predicted images

**Figure 10:**
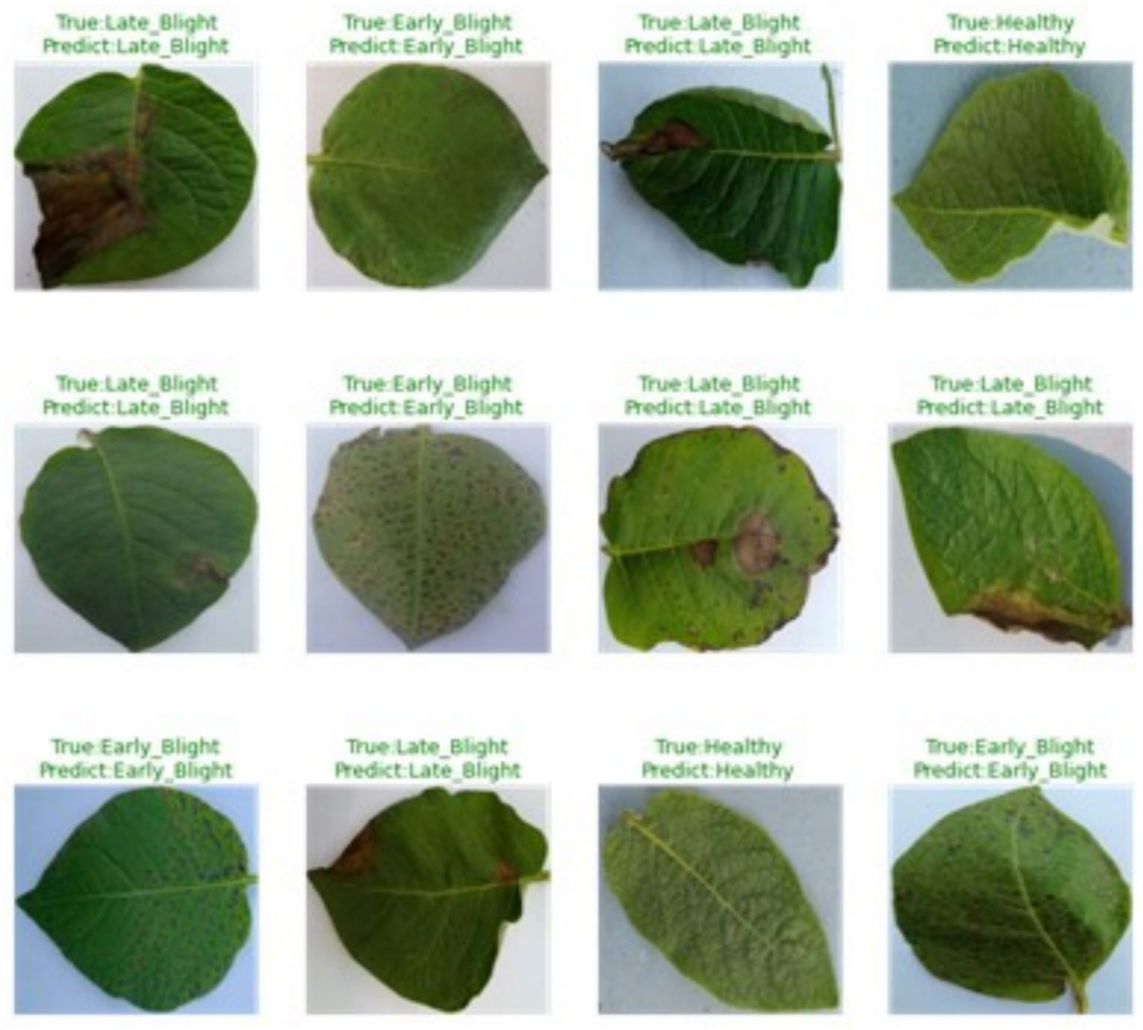
PotatoLeafNet Model performance on correctly predicted images.

The classification accuracy of the model on correctly labeled images reflects the strength of the proposed CNN framework in distinguishing between diseased and non-diseased leaf samples. A correctly identified instance refers to an image that the model assigns the appropriate label to, whether the leaf is affected or unaffected. This aspect of performance was evaluated using standard accuracy-based metrics. The model consistently delivered accurate predictions across all categories, showcasing its reliability in handling both training and unseen test samples. Its ability to differentiate between various visual patterns linked to disease manifestations underlines its robustness and generalizability. The successful identification of all leaf conditions confirms the framework’s precision and operational reliability in practical settings. Figure 10 provides a visual representation of the model’s performance in identifying each class correctly, further validating its strength in class-wise prediction and its potential for real-world application in automated plant disease assessment systems.

### 4.7 Performance measures on individual diseases prediction and classification

**Figure 11:**
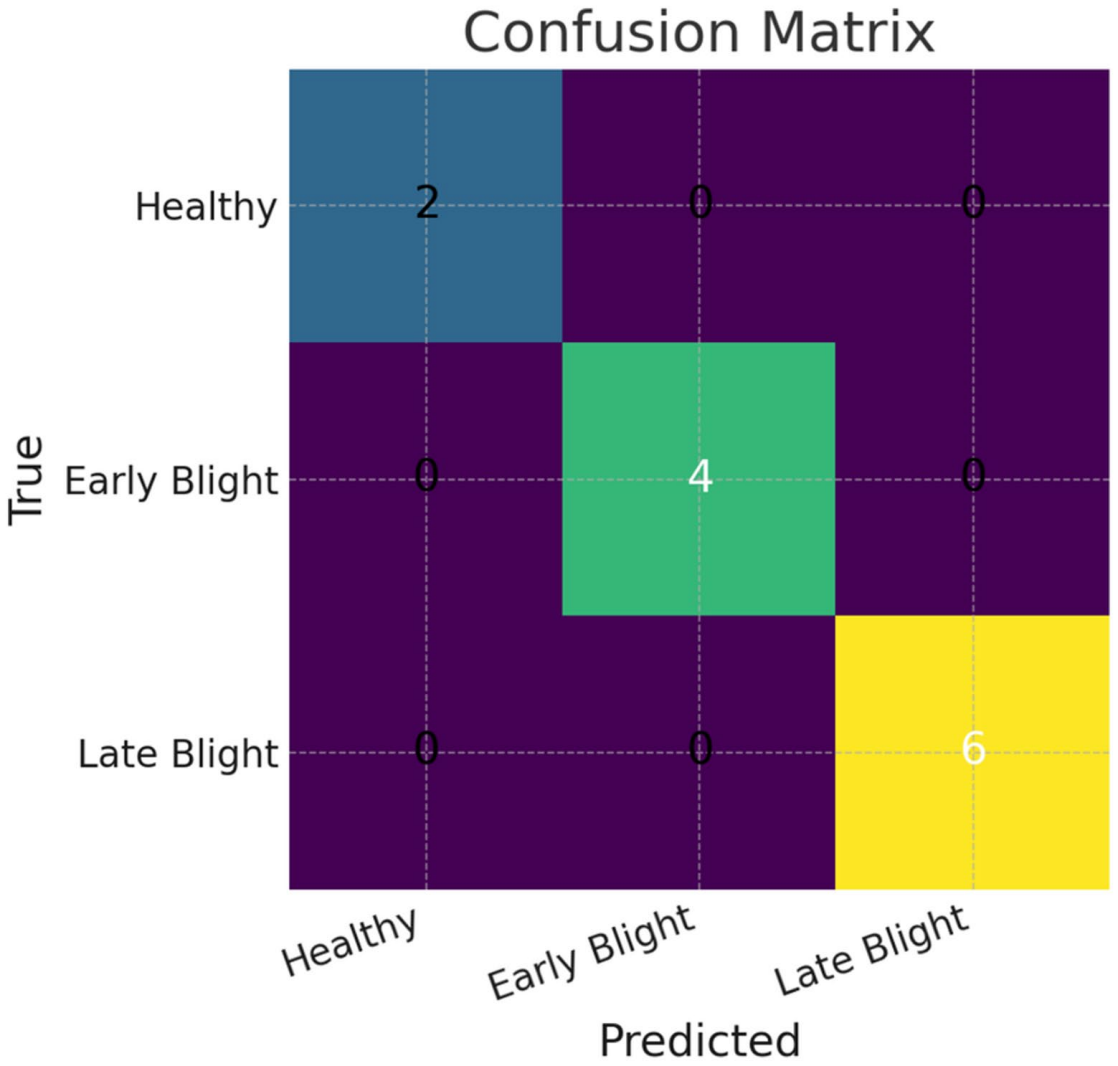
Confusion matrix for the PotatoLeafNet model on predicted individual disease classes after 100 epochs.

Collectively, these metrics provide a comprehensive view of class-wise prediction performance. Training the PotatoLeafNet model for 100 epochs yielded strong results: precision of 98.00% for Early blight, 99.00% for Late blight, and 99.00% for Healthy leaves; recall of 100.00, 99.00, and 100.00%, respectively; and F1-scores of 99.00, 99.00, and 99.50% for Early blight, Late blight, and Healthy leaves, respectively, corresponding to a macro-averaged F1 of 99.16%. Taken together, these indicators suggest that PotatoLeafNet accurately predicts and classifies potato leaf disease categories. At the same time, Table 5 indicates that Early and Late Blight can be less reliably predicted under challenging conditions such as out-of-distribution inputs, limited representativeness in the training data, noisy or ambiguous images, and potential overfitting whereas Healthy leaves are generally classified more accurately. Figure 11 presents the confusion matrix for a small held-out test subset after 100 training epochs (Early Blight = 4, Late Blight = 6, Healthy = 2). All instances were correctly identified, with no false positives or negatives, corresponding to 99% accuracy and precision/recall of >0.98 for each class on that subset. However, given the limited sample size, these perfect results should be interpreted cautiously and validated on larger, more diverse datasets to confirm generalization (Table 7).

**Table 7:** Performance measures on individual potato leaf diseases classes.

S. No	Class	Precision (%)	Recall (%)	F1-Score (%)
1	Early blight	98.00	100.00	99.00
2	Late blight	99.00	99.00	99.00
3	Healthy leaves	99.00	100.00	99.50

### 4.8 Discussion

Potato production underpins global food security, yet yields and quality are threatened by diverse foliar diseases whose early diagnosis is complicated by cultivar heterogeneity, variable symptom expression, and environmental noise, making rapid and accurate detection essential. To address this need, we propose PotatoLeafNet, a two-stage convolutional framework for automated identification of potato leaf conditions. In the first stage, a fixed sequential image-augmentation pipeline expands intra-class variability and mitigates overfitting; in the second, an 11-layer CNN with 3 × 3 kernels learns discriminative morphological and textural representations from the augmented images. Evaluated on the enhanced dataset, PotatoLeafNet achieved an overall accuracy of 98.92%, with complementary performance measures confirming its ability to correctly categorize samples. In comparative analyses, the approach outperformed representative state-of-the-art baselines and consistently predicted Late Blight, Early Blight, and Healthy classes with high reliability. By enabling precise differentiation among these categories, PotatoLeafNet facilitates timely intervention and supports evidence-based disease-management strategies in real-world agronomic settings.

## 5 Conclusion

This work introduced PotatoLeafNet, a two-stage convolutional framework that couples a fixed sequential image-augmentation pipeline with an 11-layer, 3 × 3-kernel CNN to deliver reliable detection and classification of potato leaf conditions (Healthy, Early Blight, Late Blight). Trained on an augmented and class-balanced dataset, PotatoLeafNet achieved 98.92% overall accuracy under the 100-epoch setting and maintained strong agreement between training, validation, and independent test splits; even in a constrained 10-epoch regime it sustained competitive generalization (training 88.22%, validation 86.91%, test 88.15%). In head-to-head comparisons on the same dataset, PotatoLeafNet consistently outperformed representative CNN baselines, indicating that the combination of sequential augmentation and a compact convolutional stack yields discriminative, disease-relevant representations without sacrificing computational efficiency. Practically, these attributes make PotatoLeafNet a strong candidate for field deployment in resource-limited settings (e.g., mobile or edge devices), where rapid, accurate triage can enable timely intervention, reduce losses, and support data-driven integrated pest management. While the results are robust, two limitations merit attention. First, performance was established on curated images; domain shift in truly in-situ imagery (lighting variation, occlusion, mixed infections, cultivar differences) can degrade accuracy. Second, the present evaluation emphasizes aggregate metrics; class-wise calibration, error analysis, and explainability are essential before widescale adoption. Addressing these gaps will strengthen external validity and user trust. Future work will expand training with diverse, field-acquired datasets and explicitly address domain shift through domain-generalization techniques such as style transfer and test-time adaptation; provide fine-grained diagnostics including per-class precision and recall, confusion matrices, and confidence calibration together with explainability analyses (Grad-CAM/saliency) to verify that decisions focus on pathognomonic regions; conduct ablation studies to isolate the contribution of each augmentation transform and architectural component; examine robustness under label noise and data drift across seasons and geographies; and prototype a lightweight, on-device inference stack employing batching and quantization to validate throughput and latency in real agronomic workflows, thereby advancing PotatoLeafNet from a high-performing classifier to a deployable decision-support tool.

## Data Availability

The original contributions presented in the study are included in the article/supplementary material, further inquiries can be directed to the corresponding author.

## References

[ref1] Afzaal H. Farooq M. S. Rehman A. U. Sultana S. Zafar A. Habib M. A. . (2021). Detection of a potato disease (early blight) using artificial intelligence. Remote Sens 13:411. doi: 10.3390/rs13030411

[ref2] Ahmed M. M. Aftab R. S. Hamim S. A. Abdullah-Al-Jubair M. Nandi D. (2025). “Harnessing convolutional neural networks for potato leaf disease detection: a proposed model” in Machine vision in plant leaf disease detection for sustainable agriculture. eds. Mridha M. F. Dey N. (Singapore: Springer Nature Singapore), 91–103.

[ref3] Ala’a R. (2025). Potato leaves disease classification based on generalized Jones polynomials image features. MethodsX 14:103421. doi: 10.1016/j.mex.2025.103421, 40567945 PMC12192758

[ref4] Alhammad S. M. Khafaga D. S. El-Hady W. M. Samy F. M. Hosny K. M. (2025). Deep learning and explainable AI for classification of potato leaf diseases. Front. Artif. Intell. 7:1449329. doi: 10.3389/frai.2024.1449329, 39963448 PMC11830750

[ref5] Alzakari S. A. Alhussan A. A. Qenawy A. S. T. Elshewey A. M. (2025). Early detection of potato disease using an enhanced convolutional neural network-long short-term memory deep learning model. Potato Res. 68, 695–713. doi: 10.1007/s11540-024-09760-x, 41328310

[ref6] Awal M. A. Roy K. Rahman M. M. (2019). “Potato leaf disease recognition using deep learning” in Proceedings of the 2019 international conference on robotics, electrical and signal processing techniques (ICREST). (Piscataway: IEEE), 120–125.

[ref7] Bappi I. Richter D. J. Kim K. (2025). Assessing the effectiveness of augmentation techniques in enhancing plant leaf disease classification. Smart Media J. 14, 17–25. doi: 10.30693/SMJ.2025.14.1.17

[ref8] Barman U. Choudhury R. D. Sahu D. Borah S. Das R. Roy A. . (2020). “Comparative assessment of deep learning to detect the leaf diseases of potato based on data augmentation” in Proceedings of the 2020 international conference on computational performance evaluation (ComPE); 2020 Jul 2–4, Shillong, India: IEEE. 682–687.

[ref9] Chen Y. Liu W. (2025). CBSNet: an effective method for potato leaf disease classification. Plants 14:632. doi: 10.3390/plants14050632, 40094555 PMC11902249

[ref10] Dey T. K. Pradhan J. Khan D. A. (2025). Optimized potato leaf disease detection with an enhanced convolutional neural network. IETE J. Res. 71, 1777–1790. doi: 10.1080/03772063.2025.2467761

[ref11] Ferentinos K. P. (2018). Deep learning models for plant disease detection and diagnosis. Comput. Electron. Agric. 145, 311–318. doi: 10.1016/j.compag.2018.01.009

[ref12] Fuentes A. Yoon S. Kim S. Park D. (2017). A robust deep-learning-based detector for real-time tomato plant diseases and pests recognition. Sensors 17:2022. doi: 10.3390/s17092022, 28869539 PMC5620500

[ref13] Geetharamani G. Pandian A. (2019). Identification of plant leaf diseases using a nine-layer deep convolutional neural network. Comput. Electr. Eng. 76, 323–338. doi: 10.1016/j.compeleceng.2019.04.011

[ref14] Ghosal S. Bandyopadhyay S. Sahidullah M. (2019). “Deep learning models for early blight disease detection in potato leaves” in Proceedings of the 2019 2nd international conference on advanced computational and communication paradigms (ICACCP). (Piscataway: IEEE), 1–6.

[ref15] Gupta A. Gupta R. Gupta S. (2019). Deep learning-based potato leaf disease identification using augmentation techniques. J. Agric. Sci. 7, 1–10.

[ref16] Gurucharan M. K. (2020). Basic CNN architecture: Explaining 5 layers of convolutional neural network. UpGrad Blog. Available online at: https://www.upgrad.com/blog/basic-cnn-architecture

[ref17] Hernandez-Valencia E. Ramirez-Pedraza A. Morales-Sandoval M. Sossa-Azuela J. H. Castro-Espinoza F. Aceves-Fernandez M. A. . (2020). Lossless compression for multispectral images of agricultural products based on 3D DCT. J. Appl. Res. Technol. 18, 301–309.

[ref18] Hou C. Zhuang J. Tang Y. He Y. Miao A. Huang H. . (2021). Recognition of early blight and late blight diseases on potato leaves based on graph cut segmentation. J. Agric. Food Res. 5:100154. doi: 10.1016/j.jafr.2021.100154

[ref19] Jafar A. Bibi N. Naqvi R. A. Sadeghi-Niaraki A. Jeong D. (2024). Revolutionizing agriculture with artificial intelligence: plant disease detection methods, applications, and their limitations. Front. Plant Sci. 15:1356260. doi: 10.3389/fpls.2024.1356260, 38545388 PMC10965613

[ref20] Kaur K. Kaur H. Singh M. L. Singh R. (2025). PotConvNet: an automated deep convolutional neural network-based framework for identification of potato leaf diseases. Potato Res. 68, 1–36. doi: 10.1007/s11540-025-09764-9

[ref21] Khalifa N. E. M. Taha M. H. N. Hassanien A. E. Elghamrawy S. Ghazal M. Chetouani A. . (2021). “Artificial intelligence in potato leaf disease classification: a deep learning approach” in Machine learning and big data analytics paradigms: analysis, applications and challenges. eds. Singh A. Kumar A. Sharma S. (Berlin/Heidelberg: Springer), 63–79.

[ref22] Lee T. Y. Chan C. S. Mayo S. J. Remagnino P. Ahmed F. Lim K. S. . (2020). “Health detection for potato leaf with convolutional neural network” in Proceedings of the 2020 Indo–Taiwan 2nd international conference on computing, analytics and networks (Indo-Taiwan ICAN); 2020 Feb 7–15, Rajpura, India: IEEE. 289–293.

[ref23] Li X. Zhou Y. Liu J. Wang L. Zhang J. Fan X. (2022). The detection method of potato foliage diseases in complex background based on instance segmentation and semantic segmentation. Front. Plant Sci. 13:899754. doi: 10.3389/fpls.2022.899754, 35865287 PMC9294544

[ref24] Liang Q. Xiang S. Hu Y. Coppola G. Zhang D. Sun W. (2019). PD2SE-net: computer-assisted plant disease diagnosis and severity estimation network. Comput. Electron. Agric. 157, 518–529. doi: 10.1016/j.compag.2019.01.034

[ref25] Liu J. Wang X. (2021). Plant diseases and pest's detection based on deep learning: a review. Plant Methods 17:22. doi: 10.1186/s13007-021-00722-9, 33627131 PMC7903739

[ref26] Mishra S. K. Srivastava S. (2019). Computer vision-based automated identification and classification of mango leaf diseases. J. Imaging 5:38. doi: 10.1504/IJGW.2023.13491134460466

[ref27] Nur K. N. A. Addyna N. I. Windarto A. P. Wanto A. Poningsih P. (2025). Optimization of the InceptionV3 architecture for potato leaf disease classification. JITK (Jurnal Ilmu Pengetahuan dan Teknologi Komputer) 10, 849–858. doi: 10.1016/j.jitk.2025.07.003

[ref28] Plant Village Dataset (2024). Kaggle [dataset]. Available online at: https://www.kaggle.com/datasets/mohitsingh1804/plantvillage (Accessed April 29, 2024).

[ref29] Potato Disease Types. (2025). AHDB Potatoes. Available online at: https://potatoes.ahdb.org.uk/knowledge-library/potato-disease-identification

[ref30] Potato Leaf Disease Dataset (2025). Kaggle dataset 2024. Available online at: https://www.kaggle.com/datasets/rizwan123456789/potato-disease-leaf-datasetpld

[ref31] Powers D. M. W. (2020). Evaluation: from precision, recall and F-measure to ROC, informedness, markedness and correlation. arXiv preprint arXiv:2010.16061. doi: 10.48550/arXiv.2010.16061

[ref32] Radwan M. Alhussan A. A. Ibrahim A. Tawfeek S. M. (2025). Potato leaf disease classification using optimized machine learning models and feature selection techniques. Potato Res. 68, 897–921. doi: 10.1007/s11540-024-09763-8

[ref33] Rahman M. M. Islam S. M. S. Hossain M. E. Ahmed K. (2021). Enhanced potato leaf disease classification using deep learning and data augmentation. Comput. Electron. Agric. 186:106233. doi: 10.1016/j.compag.2021.106233

[ref34] Rathod R. Shah R. Patel S. Jani A. Mehta K. Desai V. . (2020). Potato disease detection using deep learning and transfer learning. Int. J. Sci. Res. Comput. Sci. Eng. Inf. Technol. 6, 495–500.

[ref35] Rozaqi A. J. Prasetyo E. Nugroho H. A. Setiawan N. A. Santoso B. Hidayat R. . (2020). “Identification of disease in potato leaves using convolutional neural network (CNN) algorithm” in Proceedings of the 2020 3rd international conference on information and communications technology (ICOIACT); 2020 Nov 24–25, Yogyakarta, Indonesia: IEEE. 72–76.

[ref36] Salihu S. A. Adebayo S. O. Abikoye O. C. Usman-Hamza F. E. Mabayoje M. A. Brahma B. . (2025). Detection and classification of potato leaves diseases using convolutional neural network and Adam optimizer. Procedia Comput. Sci. 258, 2–17. doi: 10.1016/j.procs.2025.07.001

[ref37] Sangar G. Rajasekar V. (2025). Optimized classification of potato leaf disease using EfficientNet-LITE and KE-SVM in diverse environments. Front. Plant Sci. 16:1499909. doi: 10.3389/fpls.2025.1499909, 40385236 PMC12081437

[ref38] Sanjeev K. Ramesh B. Kumar V. Singh R. Sharma P. Verma A. . (2020). Early prediction of potato leaf diseases using ANN classifier. Orient J Comput Sci Technol. 13, 2–4. doi: 10.13005/ojcst13.0203.11

[ref39] Shabrina N. H. Indarti S. Maharani R. Kristiyanti D. A. Irmawati Prastomo N. . (2023). A novel dataset of potato leaf disease in uncontrolled environment. Data Brief 52:109955. doi: 10.1016/j.dib.2023.109955, 38125373 PMC10733095

[ref40] Shah S. K. Su’ud M. B. M. Khan A. Alam M. M. Ayaz M. (2025). PLDC-net: potato leaf disease classification network based on an efficient convolutional neural network. Eng. Rep. 7:e70178. doi: 10.1002/eng2.70178

[ref41] Sinamenye J. H. Chatterjee A. Shrestha R. (2025). Potato plant disease detection: leveraging hybrid deep learning models. BMC Plant Biol. 25:647. doi: 10.1186/s12870-025-06679-4, 40380088 PMC12082912

[ref42] Singh D. Jain N. Jain P. Kayal P. Kumawat S. Batra N. (2020). “PlantDoc: a dataset for visual plant disease detection” in Proceedings of the 7th ACM IKDD CoDS and 25th COMAD (CoDS COMAD 2020) (New York, NY, USA: Association for Computing Machinery), 249–253.

[ref43] Sunjoyo N. Nugroho N. (2022). Agriculture and food. World Bank. Available online at: https://www.worldbank.org/en/topic/agriculture

[ref44] TensorFlow Sequential Data Augmentation (2025). TensorFlow Tutorials. Available online at: https://www.tensorflow.org/tutorials/images/data_augmentation

[ref45] Tiwari D. Singh A. Sharma R. Verma S. Gupta P. Mishra R. . (2020). “Potato leaf diseases detection using deep learning” in Proceedings of the 2020 4th international conference on intelligent computing and control systems (ICICCS); 2020 May 13–15, Madurai, India: IEEE. 461–466.

[ref46] Tugrul B. Elfatimi E. Eryigit R. (2022). Convolutional neural networks in detection of plant leaf diseases: a review. Agriculture 12:1192. doi: 10.3390/agriculture12081192

[ref47] Weng L. Tang Z. Sardar M. F. Yu Y. Ai K. Liang S. . (2024). Unveiling the frontiers of potato disease research through bibliometric analysis. Front. Microbiol. 15:1430066. doi: 10.3389/fmicb.2024.1430066, 39027102 PMC11257026

[ref48] Yao X. Guan Z. Zhou Y. Tang J. Hu Y. Yang B. . (2020). “Hybrid compression algorithm for remote sensing images based on JPEG and fractal compression” in Proceedings of the 12th international conference on measuring technology and mechatronics automation (ICMTMA), Guangzhou, China: IEEE. 128–132.

[ref49] Zhang C. Wang S. Wang C. Wang H. Du Y. Zong Z. (2025). Research on a potato leaf disease diagnosis system based on deep learning. Agriculture 15:424. doi: 10.3390/agriculture15040424

